# The First Definite Lambeosaurine Bone From the Liscomb Bonebed of the Upper Cretaceous Prince Creek Formation, Alaska, United States

**DOI:** 10.1038/s41598-019-41325-8

**Published:** 2019-03-29

**Authors:** Ryuji Takasaki, Anthony R. Fiorillo, Yoshitsugu Kobayashi, Ronald S. Tykoski, Paul J. McCarthy

**Affiliations:** 1 Department of Natural History and Planetary Sciences, Hokkaido University, Kita 10, Nishi 8, Kita-ku, Sapporo, Hokkaido, 060-0810 Japan; 2 Perot Museum of Nature and Science, Dallas, Texas 75201 United States; 3 Hokkaido University Museum, Kita 10, Nishi 8, Kita-ku, Sapporo, Hokkaido, 060-0810 Japan; 4 University of Alaska, Department of Geosciences, Fairbanks, Alaska 99775 United States

## Abstract

The Prince Creek Formation of Alaska, a rock unit that represents lower coastal plain and delta deposits, is one of the most important formations in the world for understanding vertebrate ecology in the Arctic during the Cretaceous. Here we report on an isolated cranial material, supraoccipital, of a lambeosaurine hadrosaurid from the Liscomb Bonebed of the Prince Creek Formation. The lambeosaurine supraoccipital has well-developed squamosal bosses and a short sutural surface with the exoccipital-opisthotic complex, and is similar to lambeosaurine supraoccipitals from the Dinosaur Park Formation in having anteriorly positioned squamosal bosses. Affinities with Canadian lambeosaurines elucidate more extensive faunal exchange between the Arctic and lower paleolatitudes which was previously suggested by the presence of *Edmontosaurus*, *Pachyrhinosaurus*, tyrannosaurids, and troodontids in both regions. The presence of one lambeosaurine and nine hadrosaurine supraoccipitals in the Liscomb Bonebed suggests hadrosaurine dominated faunal structure as in the Careless Creek Quarry of the USA that was also deposited under a near-shore environment. It differs from the lambeosaurine dominant structures of localities in Russia and China interpreted as inland environments. This may suggest that lambeosaurines had less preference for near-shore environments than hadrosaurines in both Arctic and lower paleolatitudes.

## Introduction

Vertebrate animals in the Arctic have experienced physiological, behavioral, and morphological adaptations to survive in an extreme environment^1–3^. Rocks from the North Slope of Alaska are important for the understanding of the ecology of fossil vertebrates in the Arctic during the Cretaceous Period^4,5^. There are abundant fossiliferous exposures of the lower part of the Prince Creek Formation on the North Slope, which range from Campanian to early Maastrichtian in age^6^. The Prince Creek Formation is a non-marine succession deposited on a high-latitude, low-gradient alluvial/coastal plain. An integrated reconstruction of pedogenic processes and biota^7^ suggests that this ancient Arctic coastal plain was influenced by seasonally fluctuating water table levels and floods, and in distal areas, marine waters. The formation has yielded a diverse dinosaur assemblage that includes ceratopsids, dromaeosaurids, hadrosaurids, basal ornithopods, pachycephalosaurids, troodintids, and tyrannosaurids^4,5,8–16^.

The Liscomb Bonebed is one of the most prolific dinosaur bearing localities within this rock unit. Radiometric dating based on tephra near the bonebed indicates that the Liscomb Bonebed is early Maastrichtian with estimated ages of 71–68 Ma^17^, 69.1 ± 0.3 Ma^18^, and younger than 69.2 ± 0.5 Ma^19^. These dates are concordant with palynomorph analyses which suggest early Maastrichtian age^20,21^. The rocks were deposited at an estimated paleolatitude of 74.5° ± 7.5°^22^. The Liscomb Bonebed is from the distal area of coastal plain and is represented by lower delta plain facies^7^. Further, the stratigraphic interval containing the Liscomb Bonebed represents a series of episodic floods^19,23^ and specifically these episodic flood events created deposition by fine-grained viscous hyperconcentrated flows that transported the remains of scores of juvenile dinosaurs onto floodplains adjacent to distributary channels. The Liscomb Bonebed is characterized by high specimen density (up to 160–220 elements/m^2^) and has yielded over six thousand bones^15,24^. It is a monodominant multitaxic bonebed consisting of three theropod taxa (dromaeosaurid, troodontid, and tyrannosaurid)^7,10,25^ and hadrosaurid skeletal elements which comprises 98.5% of dinosaur skeletal elements^23^.

The Hadrosauridae is a derived clade of Hadrosauroidea and comprise two stem-sister lineages, Lambeosaurinae and Hadrosaurinae^26^. The Liscomb hadrosaurid materials were initially identified as Lambeosaurinae^20^, although osteological features to support the identification were not provided. Comparisons based on isolated cranial elements later demonstrated that the Liscomb hadrosaurs showed close similarity to the hadrosaurine *Edmontosaurus saskatchewanensis*^27^, which is now considered a junior synonym of *Edmontosaurus annectens*^28^. Since then, a general consensus formed that the Liscomb hadrosaur bones represent specimens of *Edmontosaurus*^5,23,29–34^. Recently, it was proposed that the Liscomb hadrosaur bones represent a new distinct hadrosaurine taxon, *Ugrunaaluk kuukpikensis*^15^. However, subsequent workers argued that the proposed new taxon was invalid in part because it was diagnosed on immature growth stage features preserved in the known specimens^35^. Despite of the taxonomic controversy, these studies have agreed upon the presence of a hadrosaurine hadrosaurid in the Liscomb Bonebed.

Here we report the first definitive lambeosaurine hadrosaurid fossil from the Liscomb Bonebed (DMNH 2014–12–266), represented by an isolated cranial material, a supraoccipital. The supraoccipital demonstrates that the Liscomb Bonebed contains both lambeosaurine and hadrosaurine materials. While co-occurrences of hadrosaurine and lambeosaurine are widely known in the northern hemisphere (e.g., Careless Creek Quarry^36–38^ and Jack’s Birthday Site^39^ of Montana, United States; Blagoveschensk locality^40,41^ and Kundur^42,43^ localities of southern Amur region, Russia; and Wulaga locality^44^ of northern Heilongjiang Province, China), the Liscomb Bonebed is the first to demonstrate the co-occurrence in the Arctic. Therefore, the new discovery offers an important opportunity to infer possible determinant factors of hadrosaurid taxonomic structure in the Arctic, in comparison with lower latitude regions.

## Results

### Systematic paleontology

Dinosauria Owen, 1842^45^

Ornithischia Seeley, 1887^46^

Cerapoda Sereno, 1986^47^

Ornithopoda Marsh, 1881^48^

Iguanodontia Dollo, 1888^49^

Hadrosauridae Cope, 1870^50^

Lambeosaurinae Parks, 1923^51^

### Description

**Figure 1:**
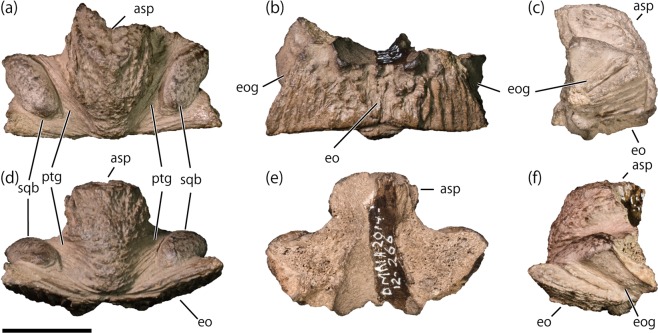
Lambeosaurine supraoccipital (DMNH 2014-12-266) from the Liscomb Bonebed. (**a**) Dorsal view. (**b**) Ventral view. (**c**) Left lateral view. (**d**) Posterior view. (**e**) Anterior view. (**f**) Right lateral view. Abbreviations: asp, ascending process; eo, articulation surface for the exoccipital-opisthotic complex; eog, exoccipital groove; ptg, post-temporal groove; sqb, squamosal boss. Scale = 2 cm.

The new supraoccipital (DMNH 2014-12-266; Fig. 1) is nearly complete but missing both anterior processes and anterodorsal end of the ascending process. Its maximum width along the posteroventral margin is 44.8 mm, which is slightly larger than those of *Edmontosaurus* sp. specimens from the Liscomb Bonebed, nearly equivalent with that of the indeterminate lambeosaurine CMN 0170^52,53^, and smaller than that of *Prosaurolophus maximus* MOR 447-8-8-7-14^52^ (Table 1). The width is also much less than the posteriorly exposed supraoccipital surfaces of adult articulated skulls of *Edmontosaurus regalis* (100.3 mm, CMN 2278), *Gryposaurus notabilis* (102.4 mm, CMN 2288), *Hypacrosaurus stebingeri* (87.1 mm, MOR 553 s; 98.0 mm, MOR 455), and *Lambeosaurus lambei* (79.5 mm, ROM 1218). Its small size may indicates that the supraoccipital (DMNH 2014-12-266) belonged to an immature individual.

**Table 1:** Selected measurements and ratios of hadrosaurid supraoccipitals.

ID	Formation	Subfamily	Taxa	Length, ventral surface (mm)	Width, posteroventral margin (mm)	Maximum height (mm)	L/W ratio (%)	H/W ratio (%)
**DMNH 2014-12-266**	Prince Creek Formation	Lambeosaurinae	unknown	12.0	44.8	24.9	26.8%	55.6%
USNM 11893	Two Medicine Formation	Lambeosaurinae	*Hypacrosaurus stebingeri*	17.9	52.8	35.3	33.9%	67.0%
UALVP 48	Oldman Formation	Lambeosaurinae	unknown	13.4	38.1	22.2	35.2%	58.3%
UALVP 53092	Oldman Formation	Lambeosaurinae	unknown	25.8	66.3	37.9	38.9%	57.1%
UALVP 53106	Oldman Formation	Lambeosaurinae	unknown	17.5	48.4	—	36.0%	—
CMN 0170	Dinosaur Park Formation	Lambeosaurinae	unknown	19.4	46.8	24.1	41.4%	51.5%
UALVP 54569	Dinosaur Park Formation	Lambeosaurinae	unknown	28.4	—	29.1	—	—
UALVP 55300	Dinosaur Park Formation	Lambeosaurinae	unknown	17.1	43.2	26.2	39.7%	60.7%
DMNH EPV 127701	Lance Formation	Hadrosaurinae	*Edmontosaurus annectens*	66.0	48.5	33.6	135.9%	69.2%
MOR 447-8-8-7-14	Two Medicine Formation	Hadrosaurinae	*Prosaurolophus maximus*	43.0	85.7	34.7	50.1%	40.5%
DMNH 22807	Prince Creek Formation	Hadrosaurinae	*Edmontosaurus* sp.	24.4	30.2	15.3	80.9%	50.5%
UAMES 4291	Prince Creek Formation	Hadrosaurinae	*Edmontosaurus* sp.	24.4	31.5	15.8	77.4%	50.1%
UAMES 12727	Prince Creek Formation	Hadrosaurinae	*Edmontosaurus* sp.	36.6	37.8	12.7	97.0%	33.6%
UAMES 21544	Prince Creek Formation	Hadrosaurinae	*Edmontosaurus* sp.	20.5	29.2	15.4	70.3%	52.7%

**Figure 2:**
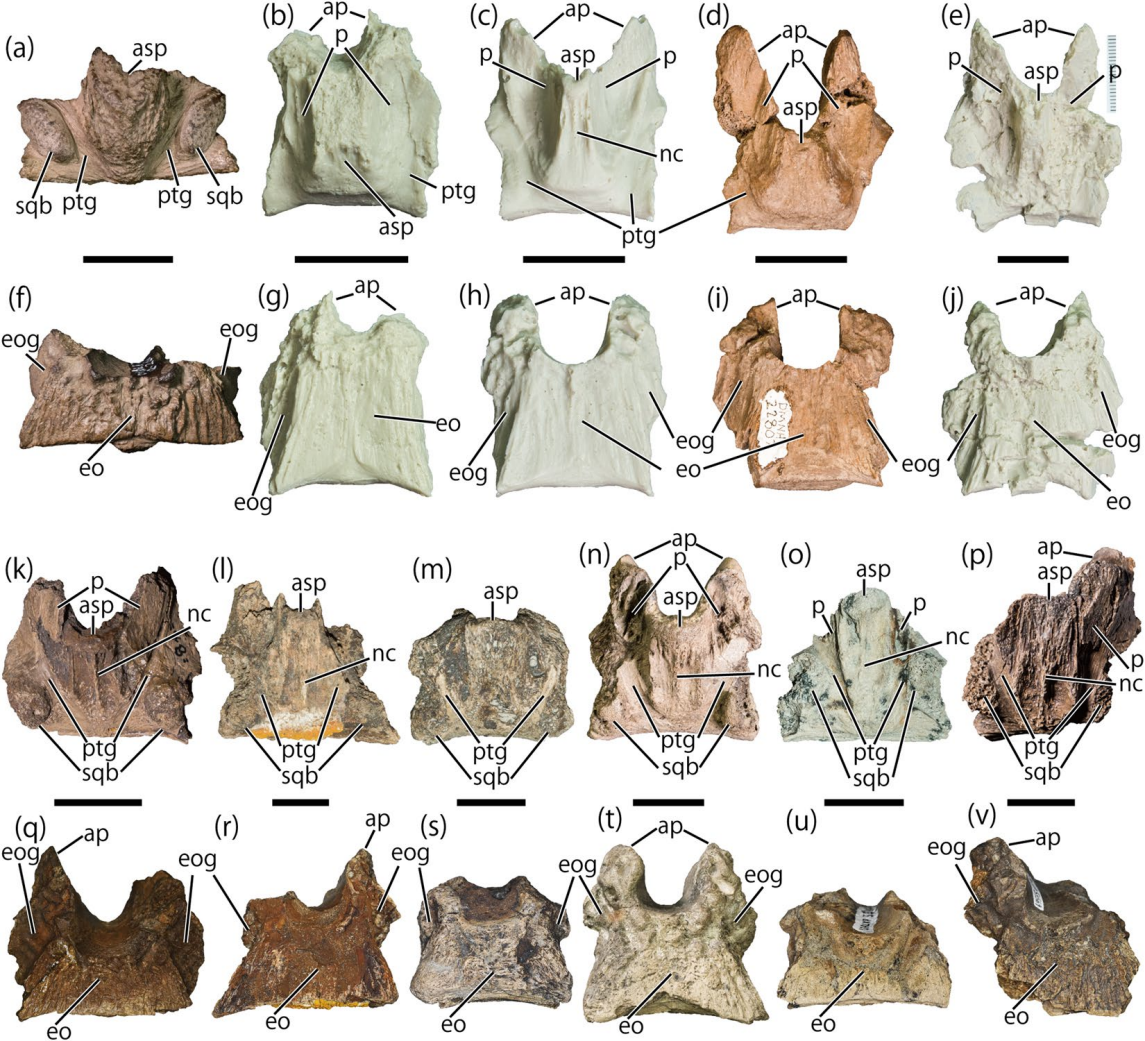
Hadrosaurid supraoccipitals. DMNH 2014-12-266 (**a**,**f**). *Edmontosaurus* sp: cast of UAMES 21544 (**b**,**g**); cast of UAMES 4291 (**c**,**h**); DMNH 22807 (**d**,**i**); cast of UAMES 12727 (**e**,**j**). Indeterminate lambeosaurines: UALVP 48 (**k**,**q**); UALVP 53092 (**l**,**r**); UALVP 53106 (**m**,**s**); CMN 0170 (**n**,**t**); UALVP 55300 (**o**,**u**); UALVP 54569 (**p**,**v**). Dorsal (**a**–**e**,**k**–**p**) and ventral (**f**–**j**,**q**–**v**) views. Abbreviations: asp, ascending process; ap, anterior process; eo, articulation surface for the exoccipital-opisthotic complex; eog, exoccipital groove; nc, nuchal crestp, articulation surface for the parietal, ptg, post-temporal groove; sqb, squamosal boss. Scale = 2 cm.

The ascending process is well-developed, taller and wider anteriorly than posteriorly, and divides the bone along the midline (Fig. 1a,d). It extends posterior to the posterior margin of the articulation surface with the exoccipital-opisthotic complex (Fig. 1a,b), unlike the anteriorly positioned ascending process of *Edmontosaurus* sp. (DMNH 22807, UAMES 4291, UAMES 12727, UAMES 21544; Fig. 2). The ascending process is convergent posteroventrally (Fig. 1a,d) as in *Hypacrosaurus stebingeri* (USNM 11893^54,55^), while those of *Edmontosaurus* sp. (DMNH 22807, UAMES 21544, UAMES 4291, UAMES 12727; Fig. 2b–e) and *Prosaurolophus maximus*^52^ are nearly parallel or divergent, and those of non-hadrosaurid hadrosauroids (*Bactrosaurus johnsoni*^56^, *Batyrosaurus rozhdestvenskyi*^57^, *Eolambia caroljonesa*^58^, *Eotrachodon orientalis*^59^) are strongly divergent posteroventrally. The dorsal surface of the ascending process is rounded (Fig. 1d) unlike the bi-lobed ascending process of *Hypacrosaurus stebingeri* (USNM 11893^54,55^). The dorsal surface is rugose and lacks the nuchal crest. On either side of the ascending process, a deep post-temporal groove^53^ runs anteroposteriorly (Fig. 1a,d) unlike in supraoccipitals of non-hadrosaurid hadrosauroids which have no distinct post-temporal groove^56–59^. The grooves are strongly divergent anteriorly as in the indeterminate lambeosaurine (CMN 0170^52,53^; Fig. 2n), differing from those of *Prosaurolophus maximus* (MOR 447-8-8-7-14^52^) and *Edmontosaurus* sp. (DMNH 22807, UAMES 4291, UAMES 12727, UAMES 21544; Fig. 2b–e) which run nearly parallel to or only slightly divergent from each other. Lateral to the groove, an anterolaterally oriented squamosal boss is present (Fig. 1a,d). The squamosal bosses are well-developed unlike in *Prosaurolophus maximus* (MOR 447-8-8-7-14^52^) and *Edmontosaurus* sp. (DMNH 22807, UAMES 4291, UAMES 12727, UAMES 21544; Fig. 2b–e). The squamosal bosses of DMNH 2014-12-266 are formed solely by the supraoccipital without participation of the exoccipital-opisthotic complex. This morphology of the squamosal boss differs from those of *Hypacrosaurus altispinus* (AMNH FARB 5248^60^), *Hypacrosaurus stebingeri* (USNM 11893^54,55^), the indeterminate lambeosaurine (CMN 0170^52,53^; Fig. 2n), and non-hadrosaurid hadrosauroids (*Bactrosaurus johnsoni*^56^, *Batyrosaurus rozhdestvenskyi*^57^, *Eolambia caroljonesa*^58^), in which the boss is also formed in part of the exoccipital-opisthotic complex.

The anteroposterior length of the ventral sutural surface is short (Fig. 1b), being 26.8% of the mediolateral width along the posteroventral margin. The ratio is much smaller than those of the Liscomb *Edmontosaurus* sp. (DMNH 22807, UAMES 4292, UAMES 21544, UAMES 12727), *Edmontosaurus annectens* (DMNH EPV 127701), and *Prosaurolophus maximus* (MOR 447-8-8-7-14^52^), but resembles lambeosaurines (Table 1). The sutural surface with the exoccipital-opisthotic complex is bowed ventrally toward the midline (Fig. 1d) as in the largest supraoccipital of *Edmontosaurus* sp. from the Liscomb Bonebed (UAMES 12727), but unlike in the smaller three. The exoccipital groove, located laterodorsal to the ventral sutural surface with the exoccipital-opisthotic complex, faces lateroventrally (Fig. 1b,c,f). The exoccipital groove is mediolaterally narrower than those of *Prosaurolophus maximus* (MOR 447-8-8-7-14^52^) and *Edmontosaurus* sp. (DMNH 22807, UAMES 4291, UAMES 12727, UAMES 21544; Fig. 2g–j), but resembles the indeterminate lambeosaurine (CMN 0170^52,53^; Fig. 2t). The anterior surface of the supraoccipital is smooth and slightly concave to form a part of the endocranial wall (Fig. 1e). However, detailed morphology of the endocranial wall is uncertain because of the missing anterior processes. The height of the supraoccipital is 55.6% of its posteroventral width (Fig. 1d,e; Table 1).

## Discussion

**Figure 3:**
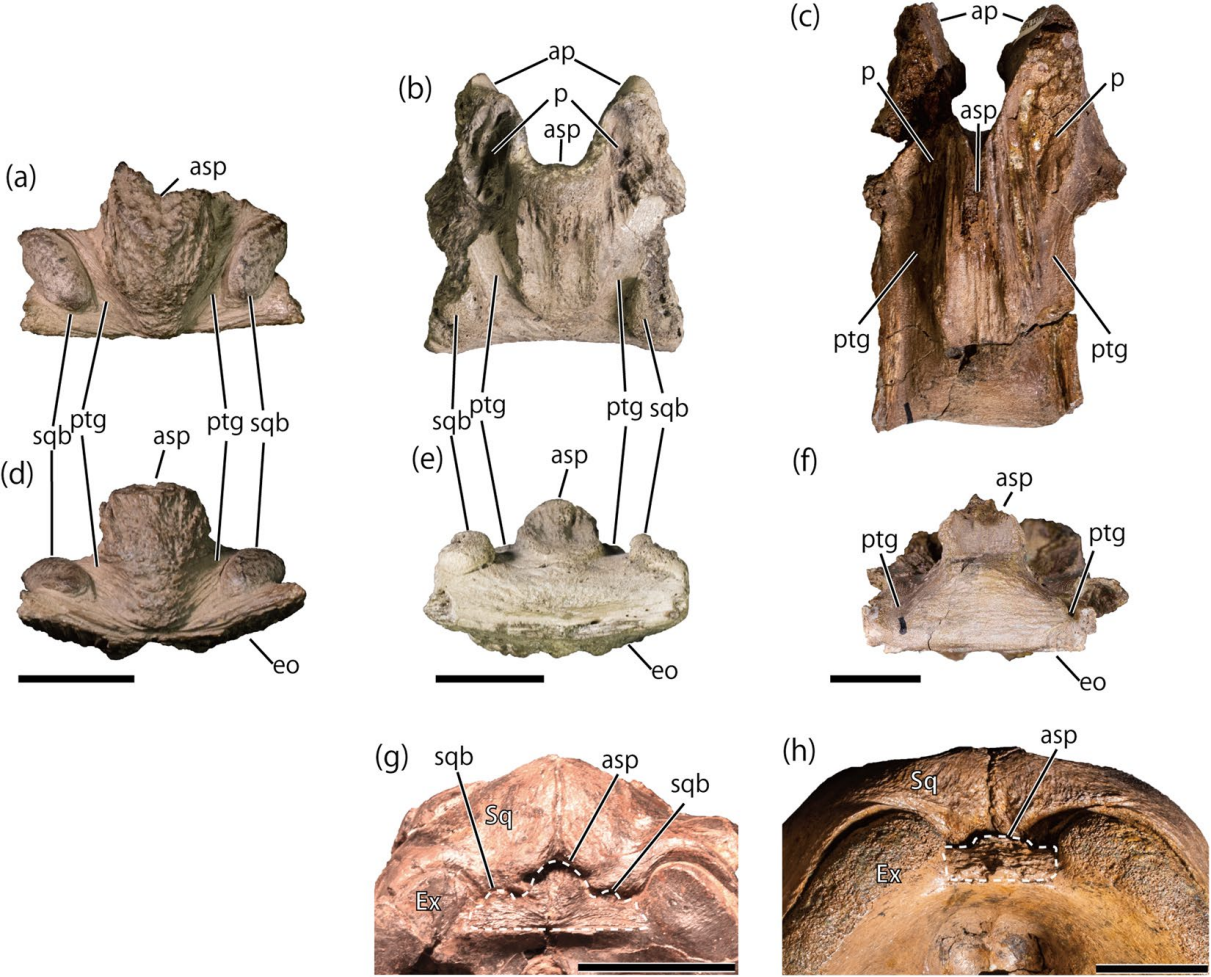
Supraoccipitals of Liscomb hadrosaurid DMNH 2014-12-266 (**a**,**d**), an indeterminate lambeosaurine CMN 0170 (**b**,**e**), and *Edmontosaurus annectens* DMNH EPV 127701 (**c**,**f**) in dorsal (**a**–**c**) and posterior (**e**–**f**) views. Posterior views of articulated skulls of *Hypacrosaurus stebingeri* (**g**) and *Edmontosaurus annectens* (**h**). Abbreviations: asp, ascending process; eo, articulation surface for the exoccipital-opisthotic complex; ptg, post-temporal groove; sqb, squamosal boss; Ex, Exoccipital-opisthotic complex, Sq, Squamosal. Scale = 2 cm (**a**–**f**), 5 cm (**g**,**h**). Dashed line represents the boundary of supraoccipital in (**g**,**h**).

The new hadrosaurid supraoccipital DMNH 2014-12-266 largely differs from those of the Liscomb *Edmontosaurus* sp. in the presence of the well-developed squamosal bosses (Fig. 2a–e) and the short exoccipital articulation surface (Fig. 2f–j; Table 2). The length of the exoccipital articulation surface is equivalent with a phylogenetic character that differentiates hadrosaurines from lambeosaurines and non-hadrosaurid hadrosauroids (degree of the caudal extension of the supraoccipital-exoccipital shelf^35,61,62^). The well-developed squamosal bosses are widely seen in lambeosaurines as well as in a few non-hadrosaurid hadrosauroids, but has never been reported in hadrosaurines (Fig. 3; Table 2). The appearance of squamosal bosses is an ontogenetic change in the non-hadrosaurid hadrosauroid *Bactrosaurus johnsoni*^56^; however, the presence of well-developed squamosal bosses in both juvenile (AMNH FARB 5461, skull length approximately 30% of the holotype MOR 549; Fig. 3g) and adult (MOR 455) individuals of *Hypacrosaurus stebingeri* suggests that the well-developed squamosal boss of DMNH 2014-12-266 is unlikely to be a result of ontogenetic variation but more likely is a taxonomic difference.

**Table 2:** List of hadrosauroid supraoccipital features.

Taxon	ID	Group	Squamosal boss	Length of the exoccipital articulation surface	Ascending process	Post-temporal grooves	Posterior exposure of the ascending process
—	**DMNH 2014-12-266**	Lambeosaurinae	Present	Less than half of the width of the posteroventral margin of the supraoccipital	Converge posteroventrally	Present, converge posteroventrally	?
*Amurosaurus riabinini*^40^	AEHM 1/232	Lambeosaurinae	Present	?	?	?	Much less than half of the width of the posteroventral margin of the supraoccipital
*Aralosaurus tuberiferus*^61^	PIN 2229	Lambeosaurinae	Present	?	?	?	Much less than half of the width of the posteroventral margin of the supraoccipital
*Charonosaurus jiayinensis*^69^	CUST JV 1251-57	Lambeosaurinae	Present	?	?	?	Much less than half of the width of the posteroventral margin of the supraoccipital
*Corythosaurus casuarius*	ROM 776	Lambeosaurinae	Present	?	?	?	Much less than half of the width of the posteroventral margin of the supraoccipital
*Hypacrosaurus altispinus*^60^	AMNH 5248 CMN 2247 CMN 8675 ROM 702	Lambeosaurinae	Present	?	?	?	Much less than half of the width of the posteroventral margin of the supraoccipital
*Hypacrosaurus stebingeri*	AMNH 5461 USNMH 11893	Lambeosaurinae	Present	Less than half of the width of the posteroventral margin of the supraoccipital	Converge posteroventrally	Present, nearly pararell to each other	Much less than half of the width of the posteroventral margin of the supraoccipital
*Jaxartosaurus aralensis*^78^	PIN 5009/1	Lambeosaurinae	Present	?	?	?	?
*Lambeosaurus lambei*	CMN 1218 CMN 2759	Lambeosaurinae	Present	?	?	?	Much less than half of the width of the posteroventral margin of the supraoccipital
*Olorotitan arharensi*^43^	AEHM 2/845	Lambeosaurinae	Present	?	?	?	Much less than half of the width of the posteroventral margin of the supraoccipital
*Velafrons coahuilensis*^79^	CPC-59	Lambeosaurinae	Present	?	?	?	Much less than half of the width of the posteroventral margin of the supraoccipital
*Acristavus gaglarsoni*^80^	UMNHVP 16607	Hadrosaurinae	Absent	?	?	?	More than half as wide as the posteroventral margin of the supraoccipital
*Edmontosaurus annectens*	DMNH EPV. 127701 ROM 53494 ROM 59786 ROM 64623	Hadrosaurinae	Absent	Half or more than the width of the posteroventral margin of the supraoccipital	Diverge posteroventrally	Present, nearly pararell to each other	More than half as wide as the posteroventral margin of the supraoccipital
*Edmontosaurus regalis*^35^	CMN 2289	Hadrosaurinae	?	?	?	?	More than half as wide as the posteroventral margin of the supraoccipital
*Gryposaurus notabilis*^81^	AMNH FARB 5350	Hadrosaurinae	?	?	?	?	More than half as wide as the posteroventral margin of the supraoccipital
*Maiasaura peeblesorum*	ROM 44770 ROM 66182	Hadrosaurinae	?	?	?	?	More than half as wide as the posteroventral margin of the supraoccipital
*Prosaurolophus maximus*^52^	MOR 447-8-8-7-14	Hadrosaurinae	Absent	Half or more than the width of the posteroventral margin of the supraoccipital	Nearly parallel	Present, nearly pararell to each other	?
*Bactrosaurus johnsoni*^56^	SBDE 95E5/29	Non-hadrosaurid hadrosauroid	Present	?	Diverge posteroventrally	Absent	?
*Batyrosaurus rozhdestvenskyi*^57^	AEHM 4/1	Non-hadrosaurid hadrosauroid	Absent	?	Diverge posteroventrally	Absent	?
*Eolambia caroljonesa*^58^	CEUM 14525 CEUM 355626	Non-hadrosaurid hadrosauroid	Absent	Less than half of the width of the posteroventral margin of the supraoccipital	Diverge posteroventrally	Absent	?
*Eotrachodon orientalis*^59^	MSC 7949	Non-hadrosaurid hadrosauroid	Absent	?	Diverge posteroventrally	Absent	?
*Levnesovia transoxiana*^82^	USNM 538191	Non-hadrosaurid hadrosauroid	Present	?	?	?	?

DMNH 2014-12-266 shows a posteroventrally convergent ascending process (Fig. 2a), which is seen only in lambeosaurines (*Hypacrosaurus stebingeri* USNMH 11893; indeterminate lambeosaurines UALVP 48, UALVP 55300, UALVP 54569; Fig. 2k,o,p), but different from a posteroventrally divergent ascending process in non-hadrosaurid hadrosauroids^57–59,63^ and a parallel or posteroventrally divergent ascending process in hadrosaurines (e.g., *Edmontosaurus annectens*, DMNH EPV 127701; *Prosaurolophus maximus*^52^) (Table 2). Additionally, the gently curved posterodorsal border of the ascending process (Fig. 1c,f) suggests anterior inclination of the posterior surface of the supraoccipital in articulation, which is a synapomorphic character of hadrosaurids^26,35^. Therefore, the combination of the four characters mentioned above (the short exoccipital articular surface, well-developed squamosal bosses, posteroventrally convergent ascending process, and anteriorly inclined posterodorsal surface of the ascending process) is unique to Lambeosaurinae (Table 2), suggesting DMNH 2014-12-266 is a supraoccipital of a lambeosaurine hadrosaur.

Isolated lambeosaurine supraoccipitals from the Oldman and Dinosaur Park formations can be divided into two morphotypes by the position of the squamosal bosses. While the squamosal bosses of the first morphotype (UALVP 48, UALVP 53092, and UALVP 53106 from the Oldman Formation and CMN 170 from the Dinosaur Park Formation; Fig. 2k,l,m,n) are posteriorly positioned, those of the other morphotype (UALVP 55300 and UALVP 54569 from the Dinosaur Park Formation; Fig. 2o,p) are anteriorly positioned, which are also seen in the Liscomb lambeosaurine (Fig. 2a). Although the Liscomb lambeosaurine shares this character with UALVP 55300 and UALVP 54569, it differs from UALVP 55300 in having posteriorly extended ascending process (Fig. 2a,f,o,u,p,v). Additionally, the Liscomb lambeosaurine differs from all other lambeosaurine supraoccipitals from the Oldman and the Dinosaur Park formations in having a rugose surface of the ascending process, the laterally completed squamosal bosses, and the ventrally bowed posteroventral margin (Figs 1 and 2). Furthermore, the Liscomb lambeosaurine also differs from penecontemporaneous lambeosaurine *Hypacrosaurus altispinus* (AMNH FARB 5248) from the Horseshoe Canyon Formation, which has weakly developed ascending process and squamosal bosses that are partly formed by the exoccipital-opisthotic complex^60^. Comparisons of supraoccipital characters with the Canadian specimens indicate that the Liscomb lambeosaurine is distinct from the Canadian specimens but shows affinities with the supraoccipitals from the Dinosaur Park Formation.

**Figure 4:**
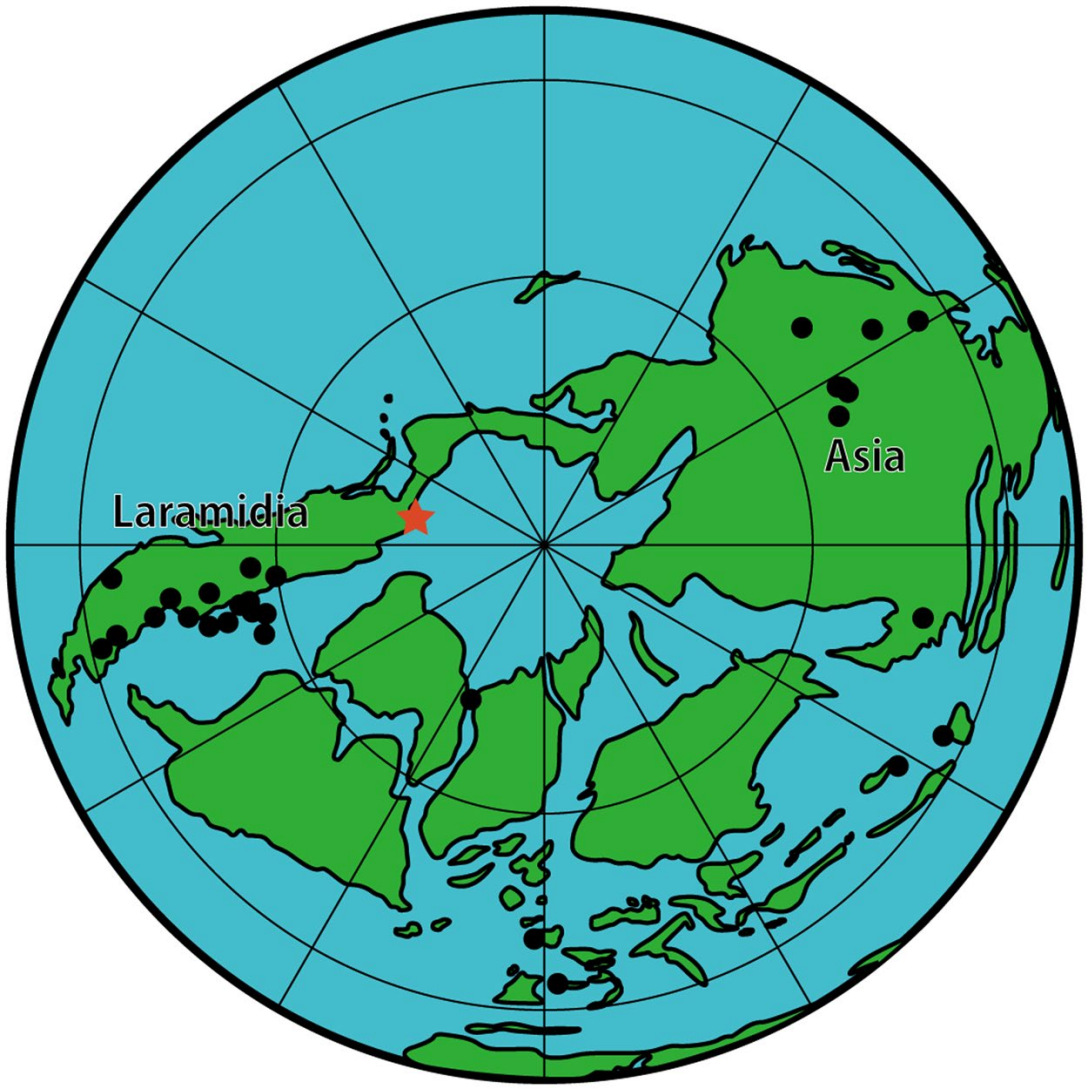
Paleogeographical records records of lambeosaurines during the Late Cretaceous. The red star represents the Liscomb lambeosaurine reported herein. Map is redrawn from Deep Time Maps^77^. The paleocoordinates are obtained from the Paleobiology Database (www.paleobiodb.org).

Previous studies suggested presence of lambeosaurine in the Arctic^29,64,65^ with no definitive descriptions of fossil materials. Russell^65^, cited by Rich and others^66^, noted occurrence of lambeosaurine from the Bylot Island of Canada, but details of the record are unknown. Russell^64^ and Gangloff^29^ mentioned possible lambeosaurine records from the North Slope of the Alaska, but the identification in the former was based on a personal communication (by John R. Horner) and the latter did not provide a specimen number or the basis for the identification. The Liscomb lambeosaurine is the first definitive occurrence of this group from the Arctic and confirms that lambeosaurines inhabited the ancient Arctic terrestrial environment. This greatly expands the paleogeographic distribution of lambeosaurines much further north than previously known from taxa such as *Hypacrosaurus altispinus* from southern Alberta, Canada (Fig. 4). At the same time, the morphological affinities with the Canadian lambeosaurines elucidate more extensive faunal exchange between the Arctic and lower paleolatitudes within North America than previously suggested, which is also supported by the presence of *Edmontosaurus*, *Pachyrhinosaurus*, tyrannosaurids, and troodontids in both regions^4,12,13,15,25,35^.

**Figure 5:**
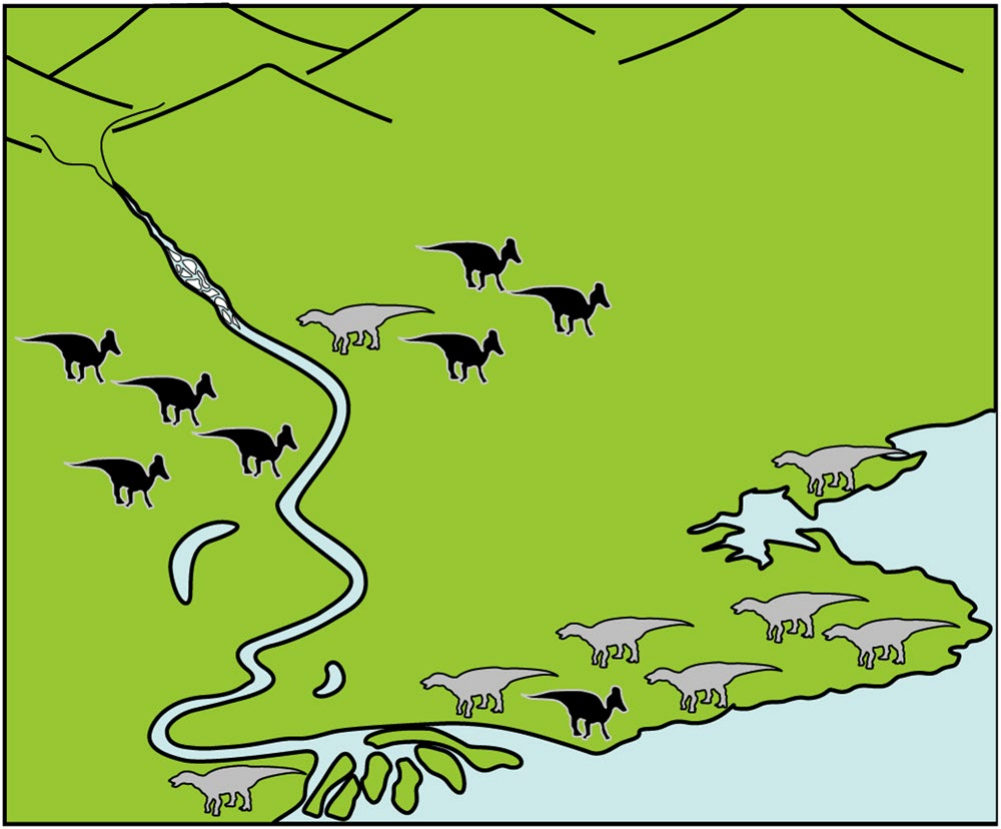
Schematic drawing of differential habitat preference between hadrosaurines and lambeosaurines.

**Figure 6:**
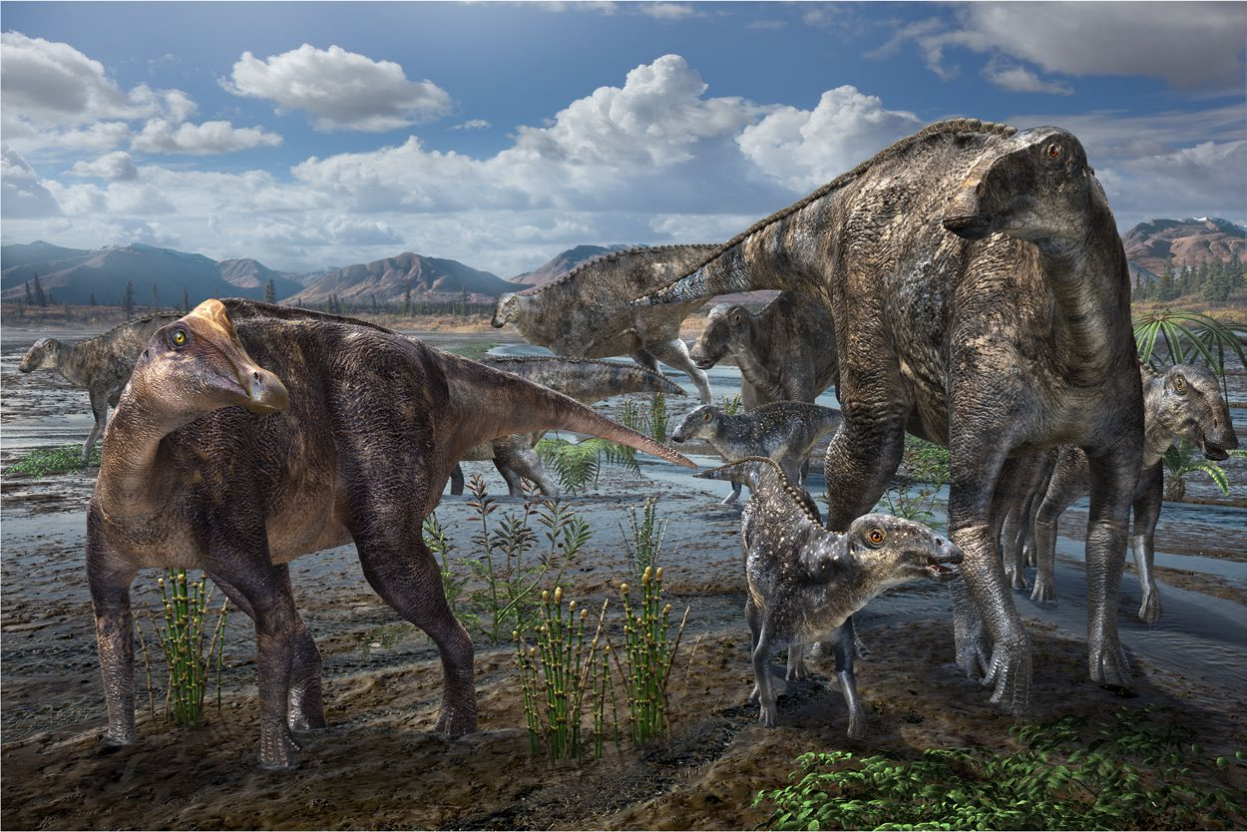
Life reconstruction of lambeosaurine-hadrosaurine co-occurrence based on the Liscomb Bonebed hadrosaurids. Artwork by Masato Hattori.

The co-occurrence of hadrosaurine and lambeosaurine supraoccipitals from the Liscomb Bonebed suggests that the validity of *Ugrunaaluk kuukpikensis* should be treated with caution because hadrosaur bones from the bonebed may consist of these hadrosaurid sub-families as well as different ontogenetic stages^35^ and, more importantly, indicates that hadrosaurine and lambeosaurine dinosaurs co-existed in the Cretaceous Arctic region. The presence of one lambeosaurine supraoccipital and eight previously reported hadrosaurine supraoccipitals^15^, as well as additional unpublished hadrosaurine specimens in the Perot Museum of Nature and Science collections, suggests numerical dominance of hadrosaurines over lambeosaurines in the ancient Liscomb region. While the hadrosaurine dominance may indicate their better adaptation to Arctic environment than lambeosaurines, hadrosaurine dominance is known from lower latitudes marine deposits^67^ and regions closer to paleoshorelines of North America^68^ and eastern Asia^34,40–44,69^, indicative of near-shore environment preferences by hadrosaurines. Consequently, the hadrosaurine dominant faunal structure of the Liscomb Bonebed, deposited in lower coastal environment, may indicate that Arctic hadrosaurids performed environment preferences similar to those in the lower latitudes (Figs 5 and 6).

## Material and Method

DMNH 2014-12-266, collected from the Liscomb Bonebed and stored in the collection of the Perot Museum of Nature and Science, Dallas, USA, was examined and described herein. Its symmetrical shape and the endocranial wall suggest that the bone is a sagittal endocranial element such as basioccipital, basisphenoid, and supraoccipital. Absences of structures present in basioccipital and basisphenoid (occipital condyle, sphenoccipital tubera, foramina for cranial nerves, basipterygoid process) leaves supraoccipital the only possible candidate. Although multiple large tetrapods are known from the Prince Creek Formation, complete exclusion of supraoccipital from the foramen magnum, suggested by the rugose sutural surface for the exoccipital-opisthotic complex, indicate that the supraoccipital does not belong to basal ornithopod^70^, dromaeosaurids^71^, pachycephalosaurines^72^, troodintids^73^, or tyranosaurids^74^. Additionally, the absence of the rostrodorsal process suggest that it does not belong to ceratopsids^75^. On the other hand, DMNH 2014-12-266 resembles the supraoccipitals of hadrosaurids in complete exclusion from foramen magnum^26^ and lambeosaurines and non-hadrosaurid hadrosauroids in presence of well-developed squamosal bosses^52–54,56,57,59^. Therefore, DMNH 2014-12-266 is identified as a supraoccipital of hadrosauroid.

Comparisons with isolated supraoccipitals of hadrosaurines from the Liscomb Bonebed (DMNH 22807 and casts of UAMES 4291, UAMES 12727, UAMES 21544, housed at the Canadian Museum of Nature), *Prosaurolophus maximus* MOR 447-8-8-7-14^52^, *Hypacrosaurus stebingeri* USNM 11893^54,55^, an indeterminate lambeosaurine CMN 0170^52,53^, and non-hadrosaurid hadrosauroids (*Bactrosaurus johnsoni*^56^, *Batyrosaurus rozhdestvenskyi*^57^, *Eolambia caroljonesa*^58^, *Eotrachodon orientalis*^59^) were made for taxonomic identification. To further investigate similarities and differences with the late Cretaceous lambeosaurines in Canada, DMNH 2014-12-266 is compared with isolated lambeosaurine supraoccipitals from the Campanian Oldman Formation (UALVP 48, UALVP 53092, UALVP 53106) and the Campanian Dinosaur Park Formation (CMN 0170, UALVP 55300, UALVP 54569). Because Xing and others^35^ argued that *Ugrunaaluk kuukpikensis* is a *nomen dubium*, we conservatively regard the hadrosaurine specimens from the Liscomb Bonebed as *Edmontosaurus* sp. as they were in prior works^5,7,23,29–33,76^.
